# Assessment on interactive prospectives of nanoplastics with plasma proteins and the toxicological impacts of virgin, coronated and environmentally released-nanoplastics

**DOI:** 10.1038/s41598-019-45139-6

**Published:** 2019-06-20

**Authors:** Ponnusamy Manogaran Gopinath, Vinayagam Saranya, Shanmugam Vijayakumar, Mohan Mythili Meera, Sharma Ruprekha, Reshamwala Kunal, Agarwal Pranay, John Thomas, Amitava Mukherjee, Natarajan Chandrasekaran

**Affiliations:** Centre for Nanobiotechnology, Vellore Institute of Technology (VIT), Vellore, 632014 TN India

**Keywords:** Risk factors, Environmental impact, DNA damage and repair

## Abstract

Recently, the concerns about micro- and nano-plastics (NPs) toxicity have been increasing constantly, however the investigations are quiet meager. The present study provides evidences on the toxicological prospectives of virgin-, coronated- and isolated-NPs on human blood cells and *Allium cepa* root tip, respectively. Several plasma proteins displayed strong affinity towards NPs and produced multi-layered corona of 13 nm to 600 nm size. The coronated-NPs often attracted each other via non-specific protein-protein attraction which subsequently induced protein-induced coalescence in NPs. In the protein point of view, the interaction caused conformational changes and denaturation of protein thereby turned it as bio-incompatible. The coronated-NPs with increased protein confirmation changes caused higher genotoxic and cytotoxic effect in human blood cells than the virgin-NPs. On the other hand, virgin-NPs and the NPs isolated from facial scrubs hindered the root growth and caused chromosome aberration (ring formation, C-mitotic and chromosomal breaks, etc.) in root of *Allium cepa*. At the outset, the present study highlights the urgent need of scrutinization and regulation of NPs use in medical applications and pre-requisition of additional studies for assessing the bio-accumulation and bio-magnification of NPs.

## Introduction

Because of modern consumption culture, plastic usage has deep routed in our daily life, thereby the global virgin plastic production escalated to a total of 8300 million metric tons (Mt) in 2015, of which around 4977 Mt waste had been generated and accumulated in natural environment^1^. Owing to the sustainability, flexibility, durability, stability, long-lasting ability, viscosity and bioavailability, the cosmetics, personal care, textile and food packaging industries incorporates plastic materials in wide range of products^2–4^. For instance, facial masks, lipstick, mascara, eye shadow, anti-wrinkle creams, soap, shampoo, conditioner, moisturizers, shower gel, hair spray, hair coloring, scrubs, toothpaste, deodorant, shaving cream, sunscreen, insect repellent, yoga pants, t-shirts, jeans, fleece, socks, running shorts, acrylic onesies, microfiber cleaning cloths, unbending plate and holders, expendable eating utensils, frothed mugs, plates, bowls^2,5,6^ and many other products ubiquitously composed of 1 to 90% of plastic. From these products as well as from indiscriminately discharged plastic polymer debris, including polyvinylchloride, polylactic acid, nylon, polypropylene, polyethylene and polystyrene, small plastic fragments called micro-plastics (cannot be decomposed or collected for recycling) are derived, enters water ways through drains and reaches aquatic environment. As a result, micro-plastics (MPs) are now pervasive in the environment and found worldwide^7^. In the environment these MPs are progressively broken into nano-plastics (NPs) due to physical, chemical and biological processes^8^.

Over the past few decades, polystyrenes a prevalent MPs and NPs being used in various day-to-day life products. These plastics can easily enter in to the cells through clathrin-coated vesicles^9^, macro pinocytosis^10^ and phagocytosis^2^ and interacts with various bio-molecules due to their expansive surface to mass proportion and smaller size. Particularly, the interaction with proteins produce polystyrene-protein complex and upon complexation, a protein coat is formed commonly alludes as corona^11^. Formation of biomolecular corona on MPs/NPs subsequently provides them a new biological identity; to escape from immune system, prolonged persistence in circulation and interfere cellular and molecular processes. Studies reported that protein corona reduces the RBC agglutination compare to the virgin-NPs^12^, successively translocate the NPs to all organs and accumulates/aggregates in liver, kidney and gut^13,14^. This uninterrupted entry of coronated-NPs in biological system could pave the way to unanticipated effects and eventually promotes them as a potential health hazard.

Recently, studies on MPs and NPs have grown exponentially revealing the discharges from the cosmetics and personal care products have potential harmful consequences towards wide range of animals and environment. For instance, the pseudocoelomate animal *Brachionus koreanus* (rotifer)^15^ and the crustaceans *Tigriopus japonicas* (copepod)^16^ showed significant reduction in growth rate and retardation, respectively upon exposure to MPs. Further, MPs displayed significant histological changes in the tissue and cellular level of blue mussel (*Mytilus edulis* L.)^17^, sea bass (*Dicentrarchus labrax*)^18^ and reduction in feeding activity in lugworm *Arenicola marina*^19^. In *Eisenia andrei* (earthworms), fibrosis, congestion and inflammatory infiltrates were observed^20^, and in microalgae namely, *Chlorella* sp. and *Scenedesmus* sp., the physical adsorption of polystyrene micro-beads inhibited the photosynthesis process^21^. Though there are indisputable evidences on the significant deformity at the cellular level and accumulation of NPs in the vital organs, the effect magnitude and the fate of micro-plastics is still uncertain. Therefore, the present study intended to assess (i) the physical changes in virgin-NPs during protein interaction, (ii) adverse effects of plasma coronated-NPs on human blood cells, (iii) toxicological impacts of virgin-NPs and the NPs isolated from cosmetics against human blood cells and *Allium cepa* root cells.

## Result and Discussion

Though MPs and NPs have constantly been released and persisted in the environment for more than 50 years, only very recently they were identified and acquainted. Since then few studies have demonstrated their adverse effects in human and environmental health. Privation of fundamental knowledge that links the physical changes of MP/NP’s to the biological macro-molecular interactions, thus triggered molecular alterations and cell toxicity is the break down to understand and evaluate the harmfulness of plastics to humans^22–24^. Therefore, in the present study, we first investigated the interactive prospectives of virgin-NPs with HSA and with human plasma followed by physical changes in NPs and proteins, secondly, tested the adverse effects of plasma coronated- and virgin-NPs on human blood cells, and finally, evaluated the cytotoxic and genotoxic effect of NPs isolated from commercial face scrubs on the human blood cells as well as *A. cepa* root tips.

### Protein corona formation on Virgin-NPs

**Figure 1:**
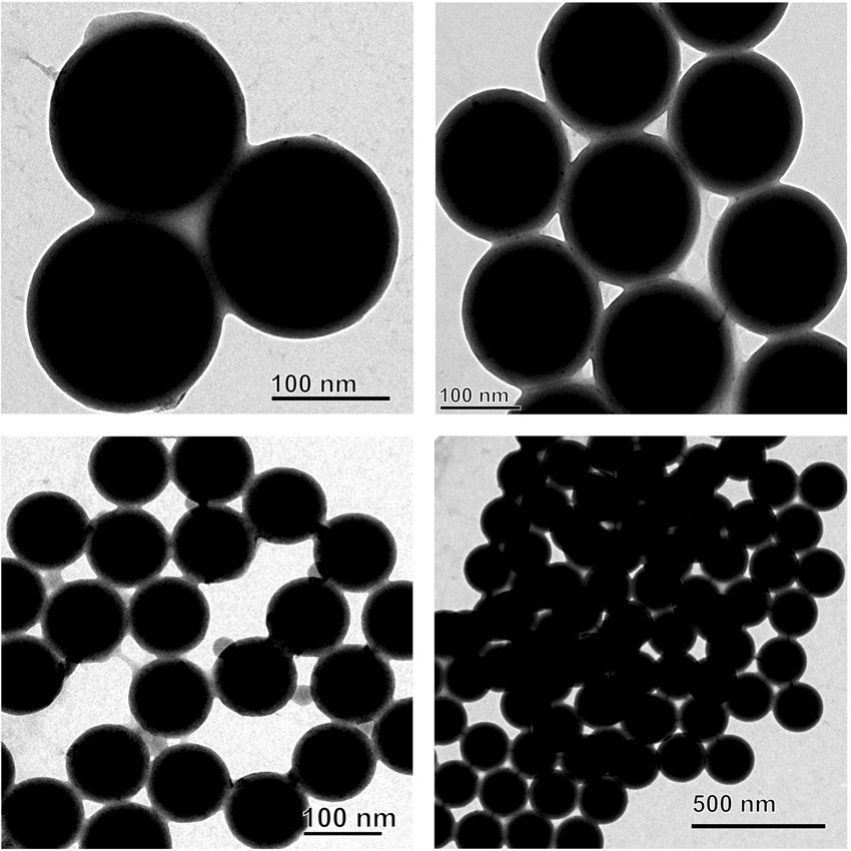
Transmission electron micrograph of nano-plastics.

**Figure 2:**
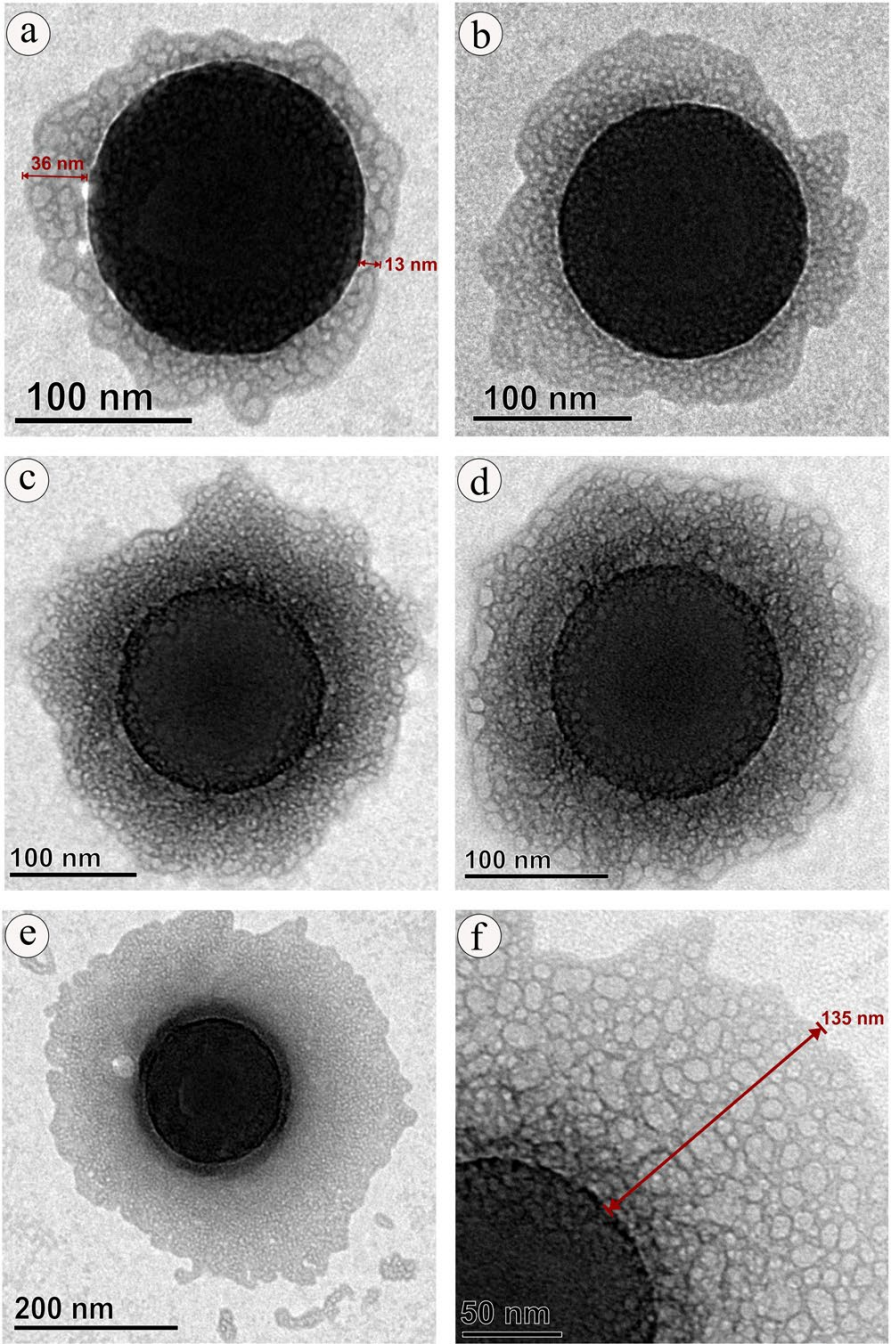
Protein corona formation on nano-plastics upon interaction with HAS.

Interaction of  virgin-NPs with HSA demonstrated three significant manifestations, such as protein corona formation, protein-induced coalescence of NPs, and secondary and tertiary structural changes in HSA. At the initial stages of the interaction, the polymeric property of NPs (Fig. 1) was disoriented and separated from each other upon HSA addition. As soon as HSA interacts with NPs, a primary cluster of HSA forms around the NPs and stabilizes by means of crowding effect^25^. This process can be repeated until the entire surface filled with HSA, resulted as protein corona (Fig. 2). In the beginning, the abundance of free HSA forms weak and dynamic interactions with NPs to set equilibrium amongst free and bound HSA. Over time, the equilibrium moves to the HSA adsorbed with higher affinity, which modifies the initial corona arrangements, thermodynamically stabilizes and brings it to an irreversible minimum energy state. The former known as soft corona consisting of loosely bound HSA due to low affinity and the latter is hard corona comprises tightly bound HSA^26^. The thickness of the corona can be determined by the secondary binders that are associated with the primary binders (HSA bound on NPs) via protein – protein interaction. Layer by layer association of secondary binders produces multi-layered corona with the size ranging from 13 nm (Fig. 2a) to 135 nm (Fig. 2f). Variation in corona size could be due to the bonding sites paucity or free protein scarcity in the solution.

Although, the binding mechanism of protein with NPs is not fully understood, it is believed that the biomolecules can binds on the NPs surfaces via non-specific attraction forces especially, Van der Waals forces or by the electric polarity alteration of approaching molecules leads to the establishment of strong bond (hydrophobic) between them or by simple dipole against dipole bonding particularly, hydrogen bond via hydrophilic chemical groups (─OH, ═O, ─NH, ═NH, ≡N)^27^ or by the spontaneous adsorption depending on the amino acid content, where the imidazole ring of histidine (His), side chains of phenylalanine (Phe) and tyrosine (Tyr), pyrrole ring proline (Pro) and isopropyl group of valine (Val) could possibly establish strong interaction with the benzene ring of polystyrene NPs^28^. Apart from these residues, aspartic acid (Asp), arginine (Arg), histidine (His), tryptophan (Trp) and glutamine (Gln) may institute the electrostatic adsorption with polystyrene NPs^29^. Considering HSA, the most abundant protein in serum composed of 18 Tyr, 31 Phe and 1 Trp, and 34 disulfide bonds that could provide rapid binding (within seconds to minutes) towards NPs and therefore, found as the major proportion of corona in the serum interaction studies^30,31^.

### Protein induced NPs coalescence

**Figure 3:**
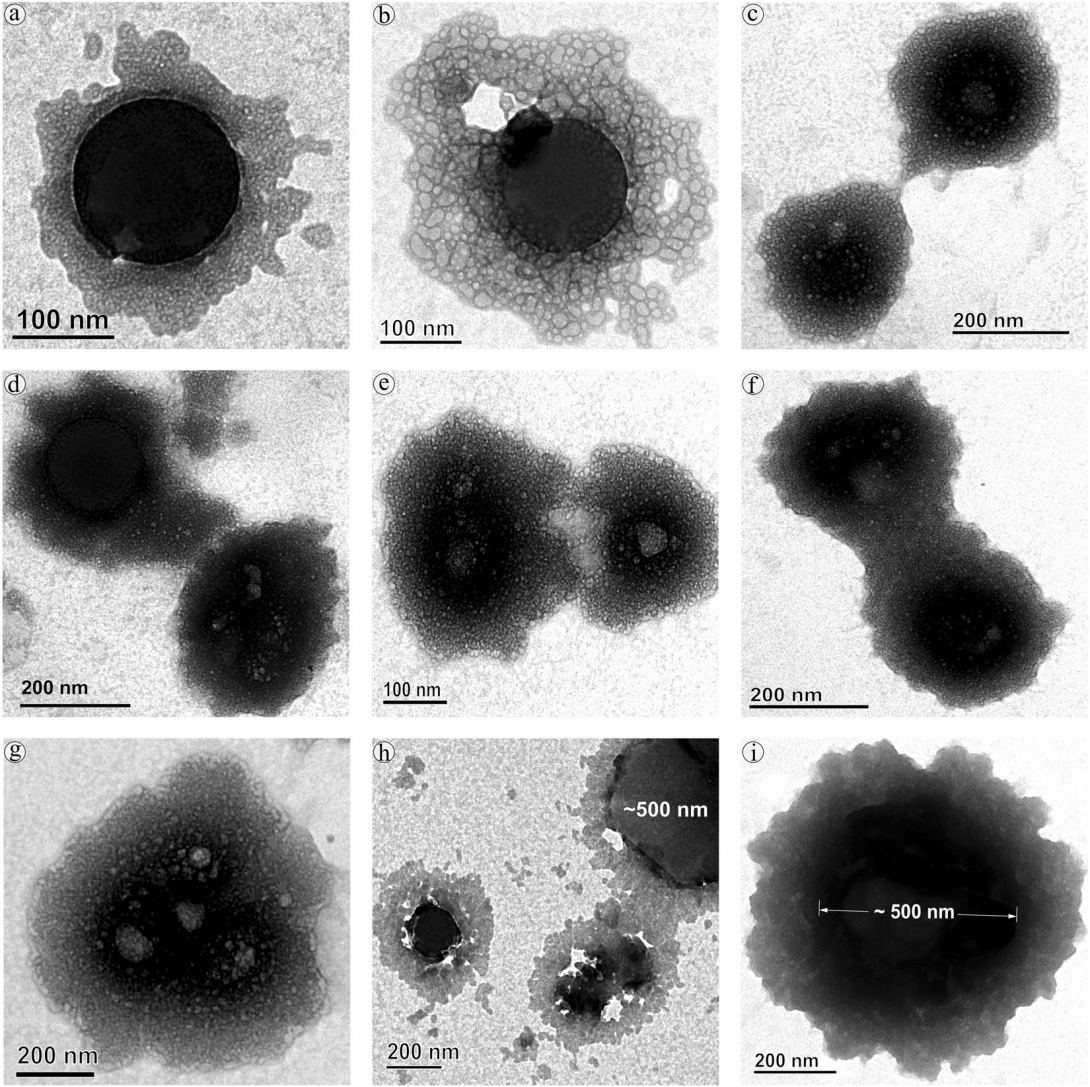
Protein-induced coalescence of nano-plastics upon interation with HAS.

**Figure 4:**
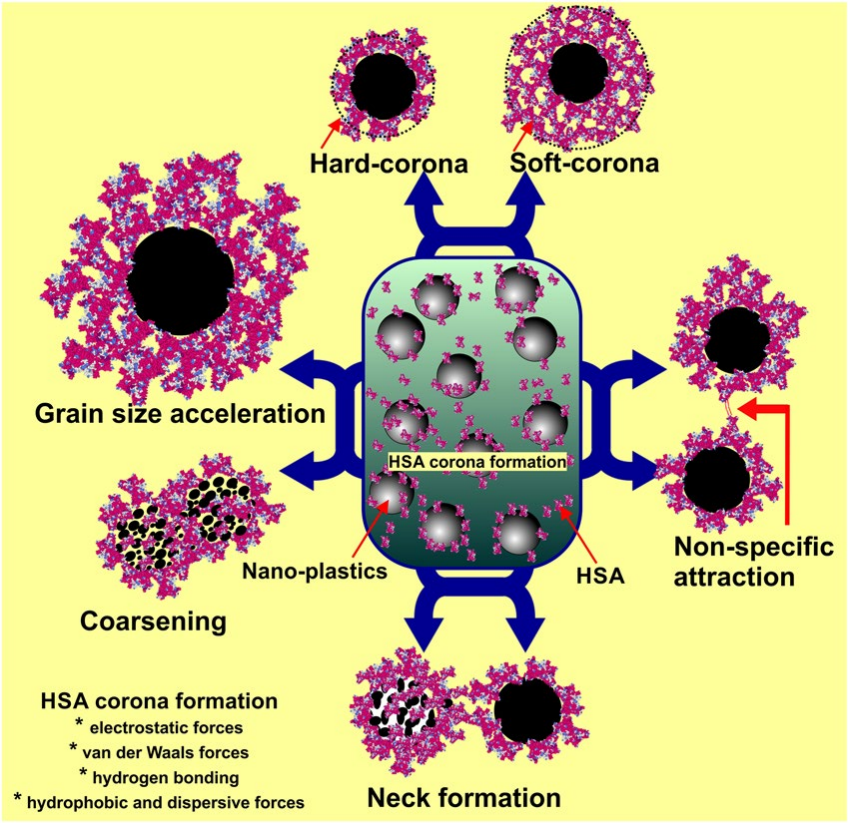
Schematic representation of HAS corona formation and HSA-induced nano-plastics grain size accelerartion.

Up till now, majority of investigation focused only on the protein confirmation and its functional properties, herein we provide first evidence on the diffusion and coalescence of NPs directed by the protein corona. Figure 3, demonstrates the approach of two coronated-NPs by means of non-specific protein-protein attraction where a thread like structure that connecting two NPs was observed (Fig. 3c), followed by corona reorientation that produces neck like bridge between two NPs (Fig. 3d,e), widening of bridge for the diffusion of NP to coalesce with other (Fig. 3f), and finally, coalesced NPs inclined to a round shape with multi-layered HSA corona (Fig. 3i). Because of the coalescence effect, there is a significant grain growth by means of successive coalescence (Fig. 3g,h) was observed in NPs. This observation clearly proves the remarkable coalescence effect of NPs upon interaction with protein (Fig. 4) that has been documented as aggregation in previous studies^13,32^. So far, many phenomenon namely, Brownian coalescence wherein collision occurs between particulates due to Brownian motion, Ostwald ripening where the particulates turns in to large ones through molecular diffusion across the intervening continuous phase, hydrodynamic coarsening via particulates interfacial velocity, coalescence-induced coalescence and diffusion-induced coalescence have been proposed for particulate coarsening effect^33^. Herein, we observed the protein-induced coalescence of NPs, where the NPs possibly dissolves during strong surface pressure produced by corona while approaching to larger coronated-NPs, diffuses across the neck like protein-bridge and coalesces with the other NPs. The TEM observations of coalescence effect revealed that three to four coronated-NPs coalesced to produce 500 nm size particles (Fig. 3g–i). Depending on the time, particle quantity and HSA concentration, the particle size can increase several fold (Fig. S3c,d).

### Protein conformation changes

**Figure 5:**
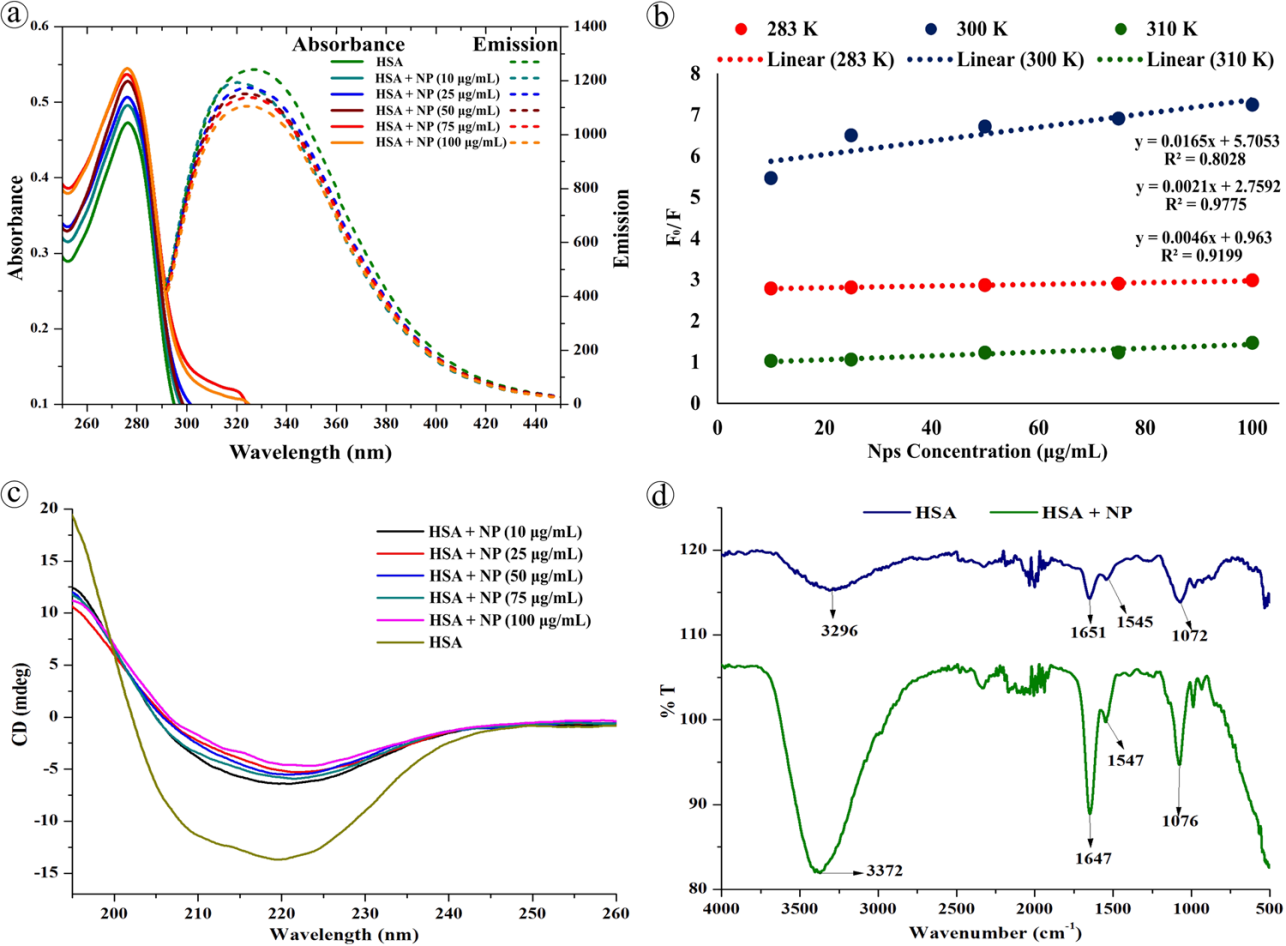
Spectroscopic characterization of HSA conformation after NPs interaction. (**a**) Abosebance and fluorescence spectra of HSA of pre- and post-NPs interaction. (**b**) Stern–Volmer plot of quenching effect at different temperature. (**c**) Cricular Dichorism spectra, and (**d**) FTIR spectra of HSA of pre- and post-NPs interaction.

On the other hand, conformational change in the protein structure was elucidated by spectroscopic and electrophoretic studies. Upon stable and continuous interaction with NPs, HSA produce hyper-chromatic effect reflecting the ground state complex formation in the micro-environment. Under UV spectrophotometry, HSA exhibited absorbance maxima ~280 nm due to π → π* transition of the tryptophan or tyrosine residues^34^. Whereas, the absorption spectra of HSA-NPs complex derived from NPs concentration 10, 25, 50, 75, & 100 μg/mL, showed gradual increase in the peak intensity at 276 nm (Fig. 5a) while at constant protein concentration, which could be due to the complexation of HSA with NP as well as secondary structure changes owing to hyper affinity. Further, there was no significant shift in the λmax observed. On contrary, the fluorescence emission property of tyrosin and/or tryptophan and/or phenylalanine residues of HSA was interfered during NPs complexation which caused variation in the intrinsic fluorescence signifying the structural changes. The λem of native HSA at 340 nm was blue shifted to 335 nm with significant quenching effect upon interaction with increasing concentration of NPs (Fig. 5a). Generally, the blue shifting of λem of protein indicates the alteration in the aromatic micro-environment and interaction of Trp residues^35,36^ with other molecules, whereas the quenching could be due to the collision of quencher molecules with the excited fluorescent residues that leads to the blocking of proton emission (collisional or dynamic quenching) or by the complex formation between the quencher and fluorophore at the ground state (static quenching)^37,38^. Apart from the excited or ground state reaction, the spectral variations can also be produced by the molecular rearrangements, energy transfer, protein conformational changes, substrate binding, subunit association and denaturation^39^. Herein, the hyper-chromatic and quenching effect of HSA upon interaction with NPs reveals the existence of static quenching as well as conformational changes in the HSA^40^. The binding parameters for HSA and NPs were assessed by Stern–Volmer (F_0_/F vs. [NP]) and double logarithmic plot (log (F_0_ − F/F) vs. log[NP]) at 290, 300, & 310 K.

1 $$\frac{F0}{F}=1+Ksv[NP]=1+Kq.\,\tau 0\,[NP]\,$$

2 $$Log\,[\frac{F0-F}{F}]=Log\,K+nLog[NP]\,$$

Wherein, F_0_ and F are the fluorescence intensity of HSA of pre- and post-NP interaction, K_sv_ is Stern-Volmer constant, K_q_ is dynamic quenching rate constant, τ0 is the life time of HSA without quencher, K is binding constant of HSA, n is number of binding sites and NP is nano-plastics in different concentration. The Stern-Volmer quenching plot presented in Fig. 5b and the Kq was found to be 0.008 × 10^13^, 0.0096 × 10^13^, and 0.0055 × 10^13^ (L mol^−1^) for 290, 300, and 310 K, respectively. From the double logarithmic eq., the K values for respective temperature were determined as 3.801, 5.726 and 1.341 L mol^−1^ and the n value was found close to be 1 denoting single binding site.

**Figure 6:**
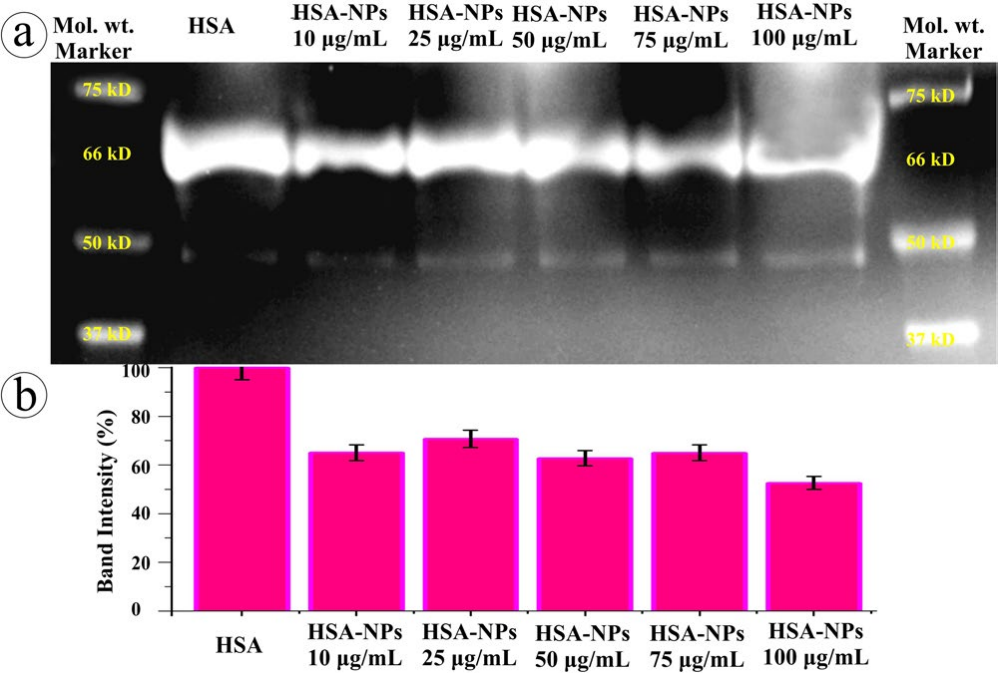
SDS-PAGE analysis of HSA conformation upon NPs interaction.

Under CD spectroscopy, the quantitative secondary and tertiary structure of HSA pre- and post-NPs complex was investigated. In which the native HSA produced two minima at 208 and 221 nm and a maxima at 215 nm corresponding to π − π* transition as well as α helix, π − π* transition for both α helix and random coiling, and β sheet, respectively^41^. Figure 5c shows the CD spectra of HSA-NP complexation, wherein a notable increase in the ellipticity at 208 and 221 nm and spectral shape changes was observed upon complexation denoting the intra-molecular H-bonding rearrangements. Complexation of HSA with different concentration of NPs showed increase in the percentage of α helix structure and reduction in β sheet percentage evidencing the additional stabilization of HSA secondary structure^42^ induced by NPs. Further, the reduction in β sheet percentage suggests the amino acid residues of polypeptide chain binding with NPs, which cause partial unfolding. Since the protein structure controls its function and drives them either as beneficial or harmful, the conformational changes in the HSA structure could eventually change their function. In order to compare the secondary structure of native and adsorbed HSA, FT-IR spectroscopy was employed. The infrared spectra (Fig. 5d) of HSA-NP complexation showed slight peak shift and drastic increase in intensity of the protein amide I band at 1651 cm^−1^ and amide II band at 1545 cm^−1^ corresponding to C═O stretch and C─N stretching coupled with N─H bending, respectively indicating the structural changes in intra-molecular bonding. Further, the strong interaction of NPs with HSA’s C─N groups was evident from the high frequency shifting of N─H stretching of amide A band at 3296 cm^−1 ^^43^ towards 3372 cm^−1^. In addition, the C─O stretching at 1072 cm^−1^ shifted to 1076 cm^−1^ upon complexation. The alterations and increase in the band intensity of amide I and II bands of HSA confirms the continuous interaction/adsorption of HSA with NPs as well as  the significant alterations in HSA confirmation^42^. Subsequently, the HSA conformation changes exhibited difference in band density among HSA of pre- and post-NPs interaction in relation to NPs concentration under SDS-PAGE (Fig. 6a). While increasing the NPs concentration, significant decrease in the band density at 67 kDa was recorded (Fig. 6b). The electrophoretic and spectral data strongly suggests that the NPs sturdily cause alterations in the HSA conformation.

### Interaction of NPs with blood plasma

**Figure 7:**
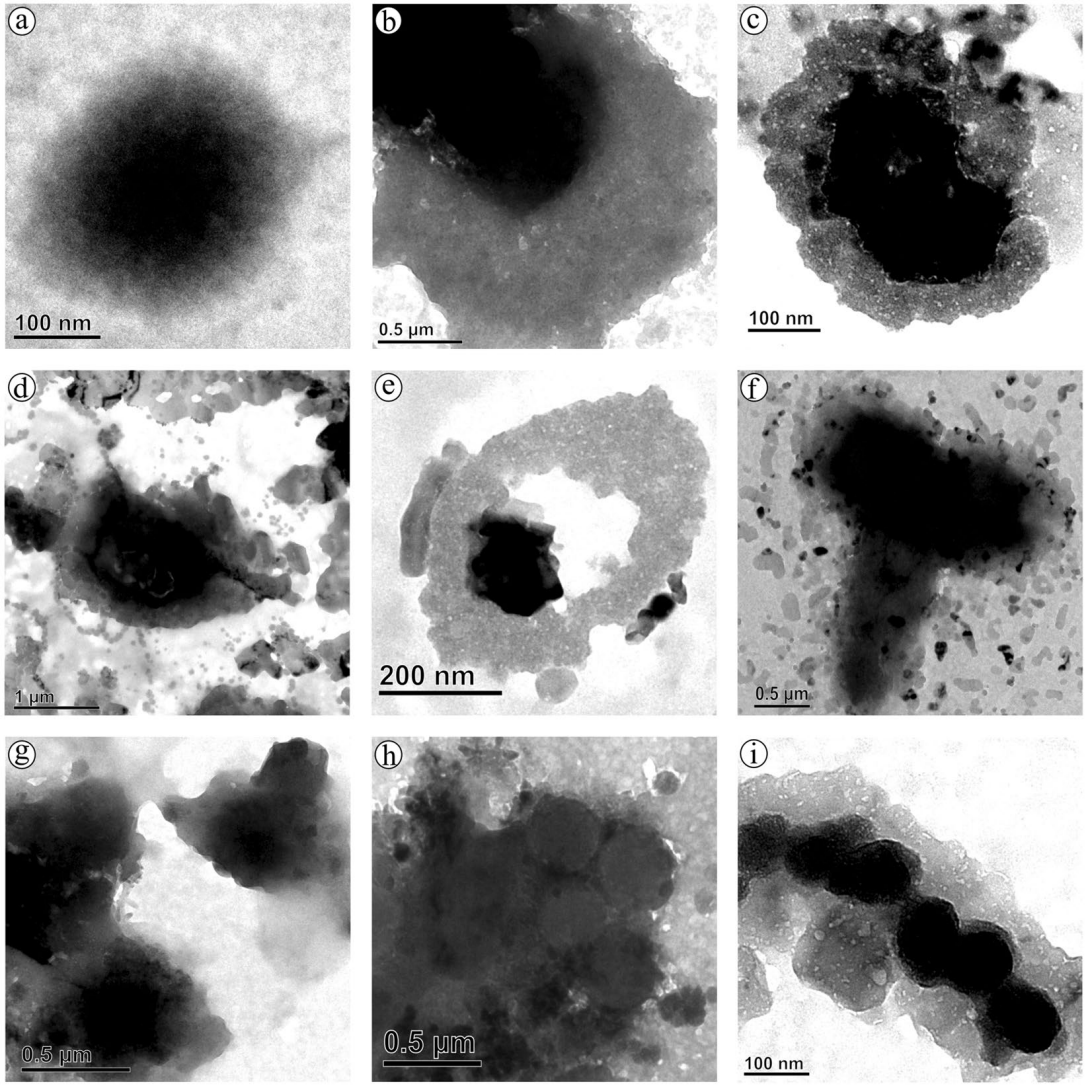
Protein corona formation and nano-plastics coalescence effect in human blood plasma.

In order to delve into the corona formation, NPs coalescence, and protein conformation effects in human body, different concentrations of virgin-NPs were introduced to human plasma. As expected, multilayered protein corona with size range of 100 to 600 nm was observed (Fig. 7). As mentioned above, increase in the corona size can possibly accomplished by the protein concentration in the medium. Since, plasma is the protein reserve of human body containing albumins as main protein mass, the corona formation could be mainly influenced by albumins followed by other plasma proteins that could probably serve as secondary binders (Fig. S4)^11,30,31,44^. During corona formation in protein mixture, protein displacement from corona due to affinity competition between proteins could occur, where the weak affinity proteins can be replaced with strong affinity proteins, called Vroman effect, which produce hard corona^45,46^. However, final protein composition in the corona can be primarily influenced by material type, size, and surface properties, as well as the protein medium composition and experimental conditions^11,47,48^.

Formation of several 100 nm size corona could comfort the cellular uptake, distribution, excretion and related toxicity of NPs inside the body. As seen in the HSA-NPs interaction, a significant increase in the grain size of NPs (upto 1 μm) was observed during plasma interaction as well (Fig. 7b,c). Such grain growth could be achieved via plasma protein-induced coalescence of multiple coronated-NPs as displayed in Fig. 7f–i. Further, these coronated-NPs were subjected to SDS-PAGE, which confirms several plasma proteins especially, albumins, globulins, fibrinogens and other proteins are adsorbed to the NPs surface^49^ and these proteins play significant roles including, maintaining osmatic pressure, molecule transport, immune response, enzyme activity and blood coagulation, respectively. SDS-PAGE analysis also demonstrated that upon increasing the NPs concentration there is a gradual decrease in the overall protein band intensity and appearance and disappearance of protein bands (Fig. S4). It appears at 50 μg/mL NPs concentration, the plasma protein interaction encounters the equilibrium amongst free and bound proteins and thus there is notable difference in the protein bands (Fig. S4) suggesting the possibility of increased proteins conformational changes.

### Human and environmental toxicity of NPs

Physico-chemical properties such as, size, shape and surface properties, and additives as well as sorbed material can influence NPs/MPs biological ramifications. Studies on fluorescence polystyrene NPs revealed that the toxicity, persistence and excretion of NPs in organisms are size dependent process. For example, in rotifers, larger NPs (>100 nm) were accumulated in gut and excreted simultaneously, whereas small NPs (<50 nm) apparently pass through the intestinal wall, entered tissues and organs through circulation, and reached extruded eggs^50^. Numerous controlled laboratory studies on MPs/NPs displayed size dependent milder to chronic conditions in various ecological targets ranging from planktonic species to filter-feeders and bottom grazers and from earthworms (*Lumbricus terrestris*) to terrestrial birds. The conditions includes, compromised immune responses in fathead minnows (*Pimephales promelas*)^32^, induced liver lesions in zebrafish (*Danio rerio*)^51^, reduced reproduction, feeding, growth rate in rotifers^50^ and *Lumbricus terrestris*^52^, embryotoxicity in sea urchin^53^ and mortality and multiple molting in brine shrimp^54^ and blockage of intestinal tract and compromised the feeding and digestion in Mediterranean seabirds^55^. Although several studies demonstrated, the accumulation and persistence of NPs in terrestrial birds, earthworms, rotifers and other organisms^56–58^, the exact damage caused by NPs or its mechanism of action were not identified, however negative effects are inevitable due to the carcinogenic plastics additives especially, phthalates, bisphenol A, brominated flame retardants, triclosan, bisphenone and organotins^59^ and sorbed pollutants^60^ that leach into cells. At the outset, it is assumed that in any organism, ingested nano-plastics will typically reach the intestine via peristaltic action, where it can be (i) incorporated with fecal matter and excreted^61^; (ii) adsorbed and entrapped across gut lining and block the intestine; or (iii) uptaken by gut enterocytes and accumulated in villi then pass through the blood stream and reaches other organs/tissues^50^ prior entering liver for recirculation into small intestine via bile^62^. It is noteworthy to mention that it has been speculated that increase in wildlife cancer might be reflected from global plastic contamination^63^ however, evidence to support or refute this claim as well as fate of NPs post-ingestion are scanty.

#### Biocompatibility of NPs in human

**Figure 8:**
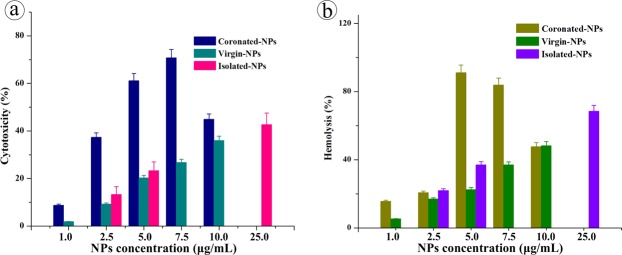
Cytotoxic and hemolysis activity of plasma coronated-, virgin- and isolated-NPs on human lymphocytes and erythrocytes.

**Figure 9:**
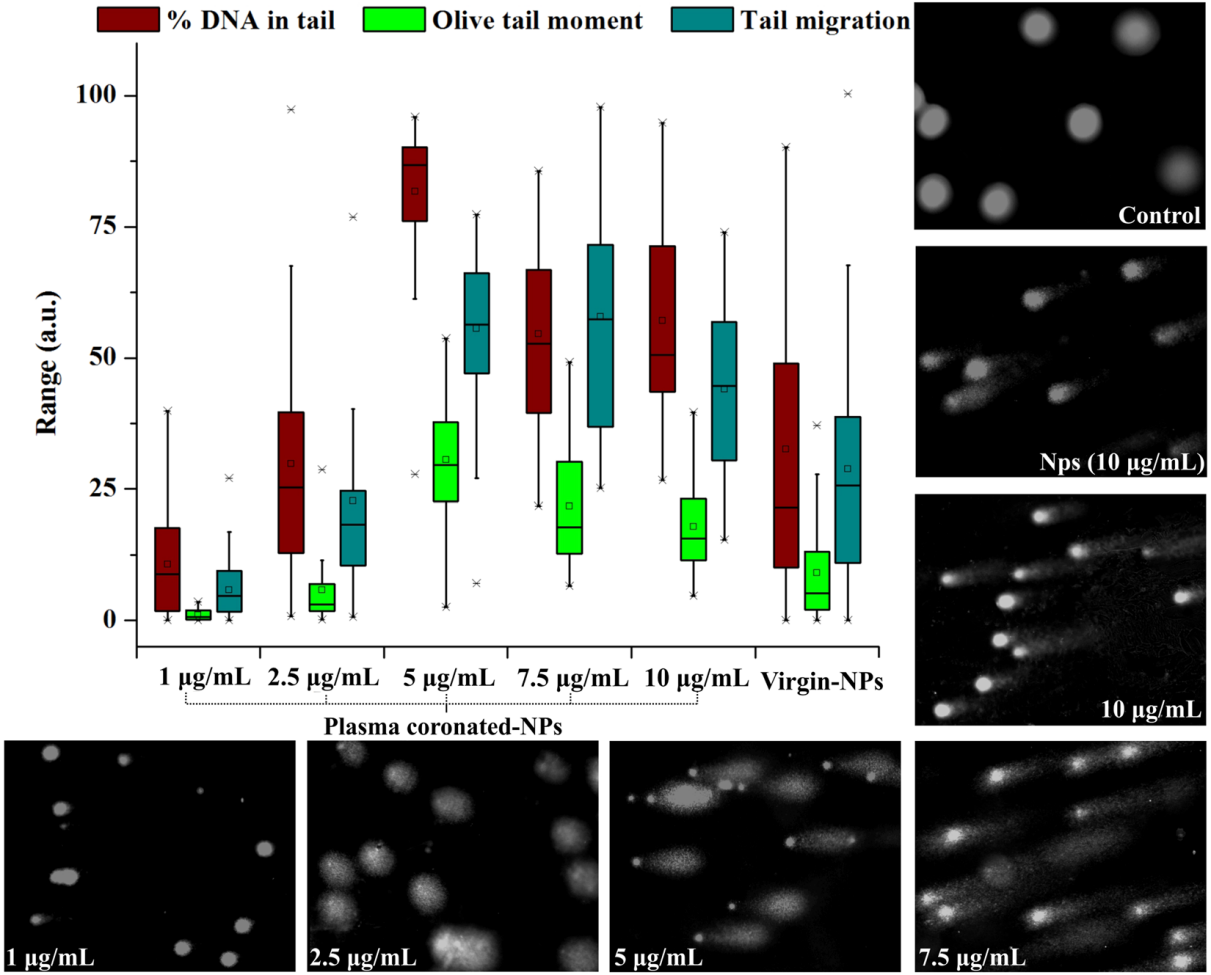
Representation of genotoxic effect of plasma coronated-NPs on human lymphocytes DNA by Comet assay.

To substantiate the toxic effects in blood stream, human lymphocytes and erythrocytes were exposed to coronated-NPs under *in vitro* condition. Wherein, the cytotoxic effect was investigated via MTT assay^64,65^ and the erythrocytes lysis was determined by hemoglobin release via spectrophotometric analysis. The percentage of cytotoxicity and hemolysis caused by coronated-NPs is presented in Fig. 8. It is witnessed that the lymphocytotoxic activity and hemolysis activity are increasing with the coronated-NPs derived from increasing concentrations of NPs and with the proteins with increased conformational changes. Five and 7.5 μg/mL coronated-NPs caused 61 and 70% cytotoxicity and 91 and 83% hemolysis, respectively on WBC and RBC as a result of higher conformational changes in plasma protein whereas virgin-NPs caused 20 and 27% WBC inhibition and 22 and 36% RBC lysis. This observation evidences that the protein coronation with increased conformation greatly influences the cytotoxicity and hemolytic activity of NPs. On the other side, isolated-NPs from face scrubs caused 25% cytotoxicity and 40% hemolysis at 5 μg/mL concentration, while 25 μg/mL showed 40 and 70% activity, respectively. The increase in the toxicity of isolated-NPs than the virgin-NPs may be due to the chemical additives carried on its surface or the existence of other toxic polymer NPs. Additionally, the genotoxic effect of coronated-, virgin- and isolated-NPs against individual lymphocytes were manifested via single cell gel electrophoresis assay. The degree of DNA damage was quantified by measuring the migration of the genetic material from cell’s nucleus and resulting DNA tail, where the cells with significant DNA damage exhibits an increased migration of DNA towards electrophoresis direction. Among the comet parameters, the most frequently used tail parameters; per cent of DNA in tail, olive tail moment and tail migration^66^ were presented in Fig. 9. Certainly, the tail moment delivers the steadiest assessments for DNA damage^66^. In the present study, an extensive DNA damage in lymphocytes was observed at 5 μg/mL concentration of coronated-NPs when compare to virgin-NPs. The observed genotoxic in lymphocytes treated with coronated- and virgin-NPs were in good correlation with the cytotoxic and hemolytic effect. The increased effect of coronated-NPs at the tested concentrations signifies the effect magnitude can be influenced by plasma protein confirmation i.e. after interaction with NPs the plasma protein turns into bio-incompatible and delivering an altered biological activity. It is noteworthy to mention that isolated-NPs showed significant genotoxic effect than virgin-NPs, however the effect is lesser than the coronated-NPs.

Studies on polystyrene NPs mediated genotoxicity (DNA damage) and chromosome aberrations in human are limited. Previously, delayed G1 phase and decreased cyclin (D, E) expression was observed in cancer HeLa cells as well as normal NIH 3T3 cells exposed to NH_2_-Polystyrene (50 nm)^67^. Similarly, DNA strand breaks in haemocytes and neurotoxic effects was observed in mussels (*Mytilus gallaprovincialis*) treated with polyethylene and polystyrene MPs^68^. Whereas in some studies, upregulation of stress regulator proteins at low concentration of polystyrene NPs was observed evidencing the oxidative stress induction, while at high concentration, gene expression was downregulated, this could be due to the damage in the antioxidative system or DNA^69^. MPs/NPs mediated oxidative damage has been reported in wide range of organisms such as lugworm *Arenicola marina*^70^, algae *Chlorella* and *Scenedesmus*^21^, Crab *Eriocheir sinensis*^71^ and zebrafish *Danio rerio*^72^. Although, there is no direct evidence portraying nano-plastics mediated genotoxicity in human, chromosomal aberrations^73^, mutations in glycophorin A^74^, sister chromatid exchange^75^, micronuclei formation^75^ and DNA single strand breaks^76^ were reported in numerous works exposed to styrene. From the present study, it is assumed that the physical damage caused by the virigin- and coronated-NPs leads to increased reactive oxygen species production and oxidative stress which ultimately cause DNA damage in blood cells similar to that of metallic nanoparticles^77^. Further, the increase in genotoxicity of coronated-NPs may be either due to the stress induced by denatured protein molecules or by coronated NPs or by the migrated styrene molecules^78^ during dissolution and coalescence effect.

#### Environmental toxicity of NPs

**Figure 10:**
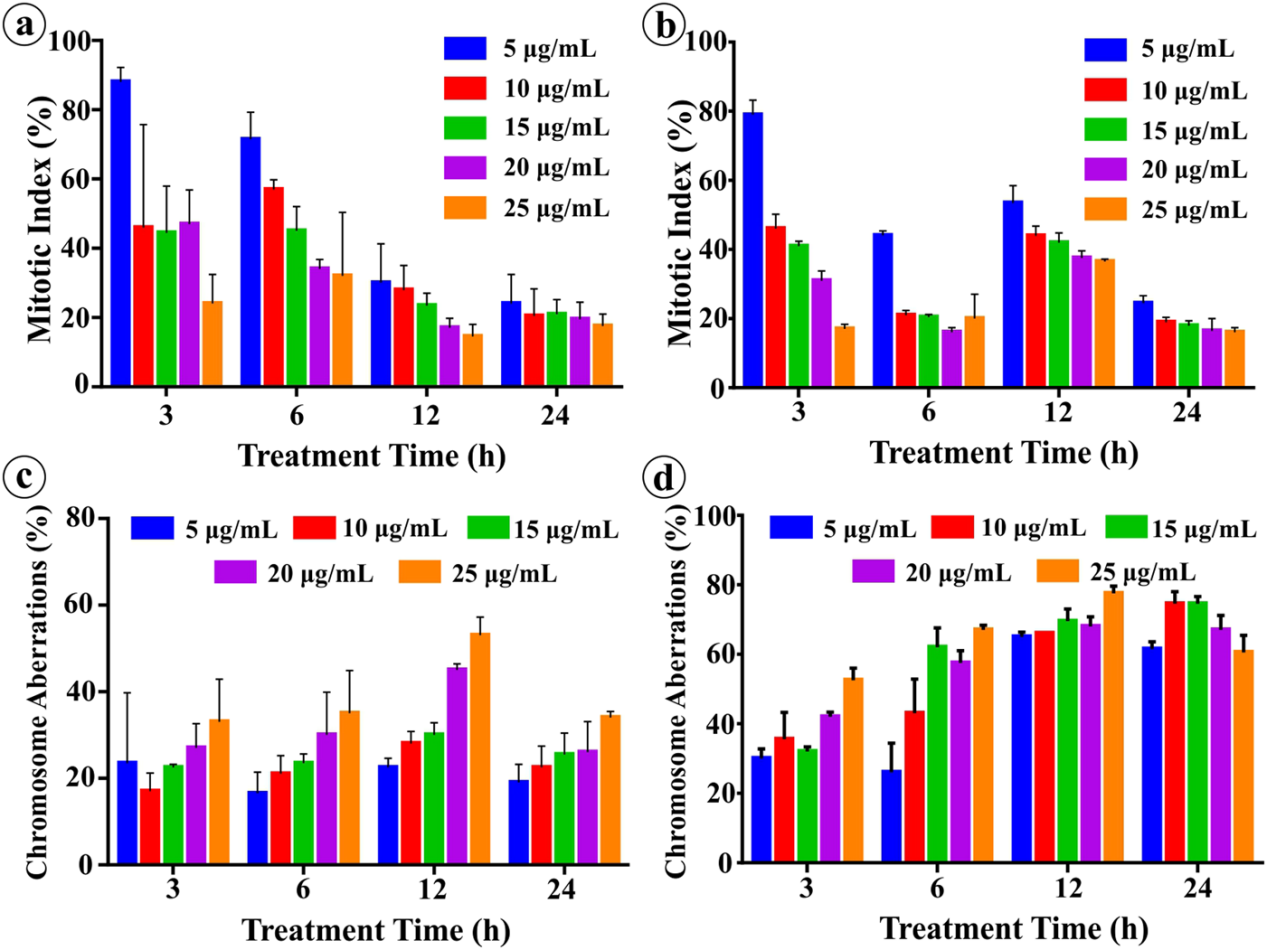
Percentage of mitotic index and chromosome aberration in the *A. cepa* root tip treated with virgin- and isolated-NPs.

**Figure 11:**
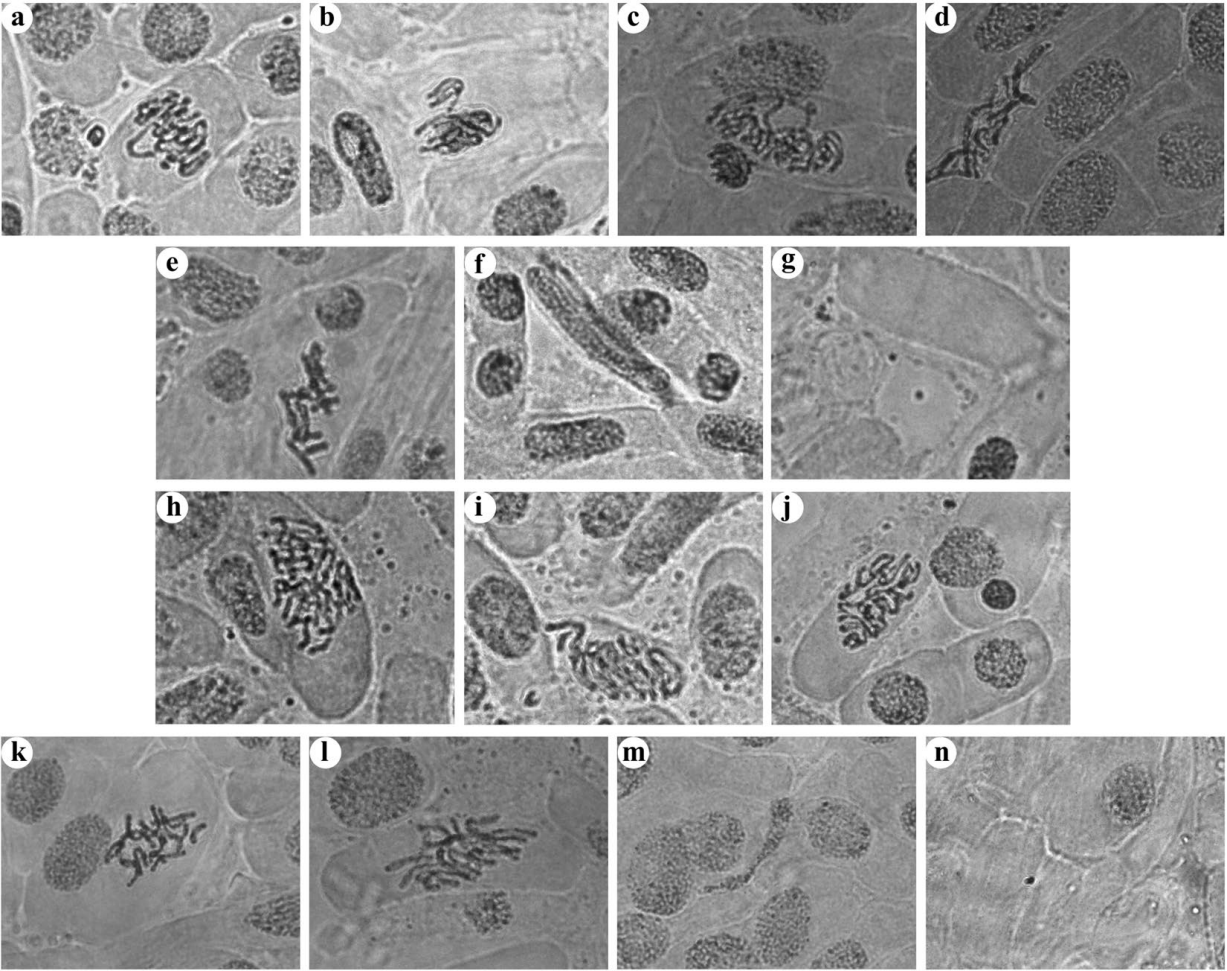
Chromosome aberrations induced by (**a**–**g**) virgin- and (**h**–**n**) isolated-NPs on *A. cepa* root tip cells. (a & i) vagrant chromosome in anaphase; (**b**,**h**) disturbed metaphase with fragments; (**c,h,j**) metaphase with ring chromosome and breaks and gap; (**d**,**l**) giant cell showing polyploidy; (**e**,**k**) C - mitosis; (f & m) strap nucleus; (**g**,**n**) enucleated cell.

On the other hand, the exposure of virgin- and isolated-NPs to onion root tips resulted in extreme decrease in mitotic index (MI) and increase in chromosome alterations (Fig. 10). Both NPs exhibited significant and dose and time dependent decrease in the mitotic index as a result of mitotic activity suppression in *A. cepa*. About, 10 μg/mL NPs caused 50% reduction in mitotic index and 25–30% chromosome aberration (CA) in 3 h treatment whereas 25 μg/mL NPs declined MI below 25% and caused 35–50% CA. Therefore, the concentrations 10 and 25 μg/mL are anticipated as sublethal^79^ and lethal^80^, respectively to *A. cepa* root cells for 3 h treatment. At these concentrations, 35% and 80% increase in oxidative stress was observed, respectively (data not presented). Further, the degree of decrease in MI and increase in CA displayed a positive correlation with increasing NPs concentration and treatment time. Additionally, we observed that depending on the treatment time the sublethal concentration can produce lethal effect to the cells. Both virgin- and isolated-NPs caused clastogenic (pulverized nucleus, ring chromosome, chromosome fragments, etc.) and non-clastogenic (C-mitosis, polyploidy, vagrant chromosomes, etc.) abnormalities in *A. cepa* (Fig. 11). Where the vagrants are the magnitudes of weak C-mitosis^81^ which lead to aneuploidy in which the chromosomes separation is unequal and the interphase nuclei is irregular^82^. C-mitosis is caused by inhibited spindle formation whereas chromosome fragmentations is caused from chromosome and chromatid breaks and these are lethal mutagenic effect^83^. The ring chromosomes are formed during chromosomes loss from the telomeric side^84^. Presence of polyploid cells attributes that the NPs can cause failure in the mitotic spindle following the C-mitosis and vagrant chromosomes formation. Alterations in the nucleic acid synthesis and protein synthesis can change the cell and/or nucleus volume producing giant strap nucleus cells. Migration of nucleus through cytomictic channels during cytomixis resulting enucleated cells^85^. The tested concentrations 5, 15, and 20 μg/mL were also caused severe mitotic inhibition signifying the increased cytotoxic, inhibitory, mitodepressive and anti-proliferative effects of virgin- and isolated-NPs. Certainly, drastic decrease in the MI suggests that there is a potential inhibition of DNA synthesis, seizure of mitotic phases and deceleration of cell progression. Apart from the prominent cytological aberrations presented, lesions, stickiness, polyploidy, hypoploid, chromosome bridges, chromosome coagulation, etc. were also detected in the root tips. The observed results clearly demonstrate the ability of NP/MPs to interrupt the nucleic acid metabolism, thereby affecting the DNA and protein synthesis, that results in an array of abnormalities. This study further provides valuable informations in relation to possible mutagenic effects of NPs on other plants and mammals. The observed abnormalities in cells demonstrates that the NP/MPs in the environment may possibly affect the mitotic spindle as well as chromosome structure of plant and animal cells when the concentration reaches the threshold level, and therefore it has to be regarded as hazardous substance.

**Figure 12:**
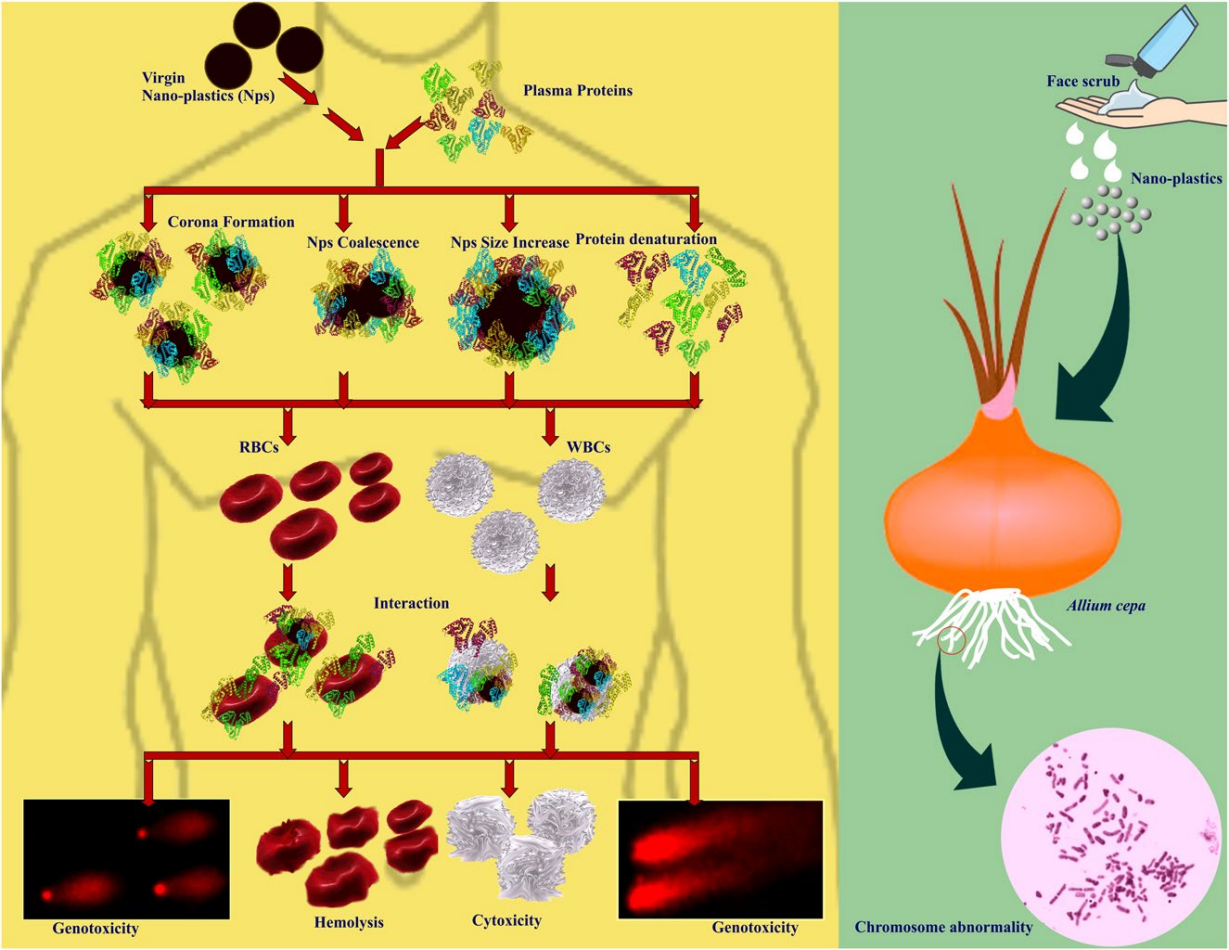
Schematic illustration of entry and fate of nano-plastics in human and environment.

At the outset, the present study provides direct evidences on; (i) multilayered corona formation on NPs during protein interaction and corona mediated protein-induced coalescence of NPs, while, from the protein point of view, the interaction caused conformational changes and denaturation of protein thereby turning it as bio-incompatible, (ii) the plasma coronated-NPs with increased protein structural alterations could cause extended cytotoxic and genotoxic effects on blood cells in compare to the virgin-NPs (Fig. 12), (iii) NPs released from cosmetics and other products could interrupt the nucleic acid metabolism; consequently affects the DNA and protein synthesis (Fig. 12). It is expected that the NPs with less/un-altered protein corona could inevitably provide a new biological identity to NPs and MPs, eventually promoting them to interfere with biological pathways and conceals them form immune response. As mentioned above, protein secondary and tertiary structural changes and deformation due to the NPs interaction and corona formation may turn the complex as immunogenic (immunogenic epitope generation) which could possibly induce the autoimmune reaction^86,87^. Recently, there has been increasing research interest in the potential application of NPs various medical applications, especially as drug delivery system^88,89^. In this circumstance, the present study highlights the urgent need for studying the adverse effects of NPs of different polymer type against human, plant and other animal systems using systematic as well as meticulous approaches, in addition to assessing the impacts of coronated-NPs on the immune system, vital organs and tissue cells and related defense response.

## Methods

### Nano-plastics samples

Polystyrene nano-plastics (Catalog # 108821-10) (NPs) with the size range of 100 nm were purchased from Corpuscular Inc., NY, USA. On the other hand, NPs (~100 nm) (Fig. S1) was isolated from commercial facial scrubs using sequential filtration technique^8^ and used for further studies. Detailed isolation procedure is presented in the Supplementary Information.

### Interaction of virgin-NPs with human serum albumin and spectral analysis

In the neutral buffer system, 0.1% of human serum albumin (HSA, Sigma–Aldrich, India) was allowed to interact with variable concentrations (10, 25, 50, 75, 100 μg/mL) of virgin-NPs for 4 h at 110 rpm in an incubator shaker. After incubation, HSA-NPs complex was separated and subjected to spectral property analysis where the changes during interaction was monitored between 200–500 nm under double beam spectrophotometer (U-2910-HITACHI, Japan). Similarly, the intrinsic emission spectra of protein after interaction with NPs at different temperatures 283 K, 300 K, & 310 K were recorded with excitation and emission wavelengths of 280 nm and 340 nm, respectively using a spectrofluorimeter (JASCO FP-8300, Japan) equipped with thermostatically peltier compartment operating with the scan speed of 200 nm/min. Further, the modifications in the secondary structure of protein after NPs interaction was measured at far UV CD spectral range (200 to 260 nm) using Circular Dichorism Spectropolarimeter (JASCO J-715, Japan) and the element of secondary structure protein were analyzed using an in build software provided by the JASCO manufacture (Tokyo, Japan). Finally, the HSA-NPs interaction was analyzed in IRAffinity-1 FT-IR spectrophotometer (Shimadzu, Japan) between the wavenumber 4000 to 500 cm^−1^. All the experiments and analysis preformed in this study were triplicated.

### Interaction of virgin-NPs with blood plasma

Human blood was collected from five healthy male volunteers (25 to 35 year old, non-alcoholic and non-smokers) through venipuncture into heparinized vacutainers followed by plasma, erythrocytes and lymphocytes were separated and used for further experiments. All the experiments were performed in accordance with relevant bioethics guidelines and regulations and approved by Institutional Ethical Committee for Studies on Human Subjects (VIT/IECH/014/01.2015). The healthy male volunteers gave informed consent. Similar to HSA studies, five different concentrations of NPs (10, 25, 50, 75, 100 μg/mL) were allowed to interact with 3 volumes of blood plasma of each individual at room temperature for 4 h, respectively. After incubation, the samples were subjected to HR-TEM, SDS-PAGE and toxicity analysis.

### SDS-PAGE analysis

HSA and plasma samples of post-NPs interaction were subjected to SDS-PAGE analysis using a 5% stacking gel and 10% separating gel under Mini-PROTEAN tetra cell system (Bio-Rad Laboratories, Inc., USA). Electrophoresis was performed according to the manufacturer’s instructions and the protein was visualized by Coomassie blue staining^90^.

### Microscopic examination

Physical changes in the virgin-NPs interacted with HSA and plasma was examined under HR-TEM (Technai, G2 20 Twin, FEI, USA) as well as HR-SEM (Carl Zeiss Evo 18 SEM, Germany) by fixing a thin film of HSA/plasma-NP complex on copper grid. Prior to SEM analysis, the sample was coated with gold using sputter coater.

### Cytotoxic effect of virgin-, coronated-, and isolated-NPs, *in vitro*

Lymphocytotoxic effect of NPs was assessed by evaluating metabolic activity of cells via MTT (3-(4,5-dimethylthiazol-2-yl)-2, 5-diphenyl tetrazolium bromide) assay. Briefly, 100 μL of freshly isolated lymphocytes (10^6^ cells/mL) were seeded in flat bottom 96-well plate containing serum free RPMI-1640 medium. Later, cells were treated with 100 μL of plasma-NPs complex containing 1, 2.5, 5, 7.5 and 10 μg of NPs derived respectively from 10, 25, 50, 75 and 100 μg/mL of NPs-plasma interaction. In similar fashion, virgin-NPs dissolved in PBS were also used for the treatment. On the other hand, the toxicity of isolated-NPs was tested using 2.5, 5 and 25 μg/mL concentrations. After 24 h incubation at 37 °C, 20 μL of MTT (5 mg/mL) was added and incubated for 4 h, then 10% of DMSO was added to the wells and kept for 20 min. Optical density at 590 nm was measured on ELISA plate reader (BioTek, PowerWave XS2, USA).

### Genotoxic effects of NPs *in vitro*

Lymphocytes isolated from the volunteers were treated with plasma coronated-NPs, virgin-NPs and isolated-NPs as above for 24 h at 37 °C. The DNA damage was assessed via comet assay using established protocol with slight modifications^64,65^. Detailed procedure is presented in Supplementary Information.

### Hemolysis activity of NPs

*In vitro* hemolysis activity of plasma coronated-, virgin-, and isolated-NPs were assayed according to Lin and Haynes,^91^. Briefly, isolated erythrocytes were washed twice and diluted in 50 mL of PBS. About 200 μL of erythrocyte suspension was transferred to PBS (700 μL) containing 1, 2.5, 5, 7.5 and 10 μg of NPs and incubated overnight at 37 °C. After incubation, the samples were centrifuged at 10000 g for 5 min and the supernatants were transferred to 96 well plate. The absorbance values at 570 nm were recorded using a ELISA plate reader (BioTek, PowerWave XS2, USA). Percentage of hemolysis was calculated by following formula;

$$ \% =\frac{{\rm{Sample}}\,{\rm{absorbance}}-{\rm{Negative}}\,{\rm{control}}\,{\rm{absorbance}}}{{\rm{Positive}}\,{\rm{control}}\,{\rm{absorbance}}-{\rm{Negative}}\,{\rm{control}}\,{\rm{absorbance}}}\times 100$$

### Toxicity study in *Allium cepa*

Bulbs of *A. cepa* were allowed to grow in ultrapure water for 2–3 days at room temperature for achieving 2–4 cm root growth. The bulbs were transferred to ultrapure water containing 5, 10, 15, 20 and 25 μg/mL of virgin-NPs. Similar set of treatment was performed with isolated-NPs. For control, separate set of bulbs were maintained in ultrapure water. At 3, 6, 12 and 24 h interval, root tips were washed (ultrapure water), hydrolyzed (1 N HCl) at 60–70 °C for 15 min. Thin sections of root tips were made and stained with aceto-orcein^92^, prior to microscopic examination (Detailed procedure is presented in Supplementary Information).

## Conclusion

Since the knowledge about biomolecule mediated transformation of NPs are scanty, it is very much required to understand the NPs behavior with biomolecules and its toxicity using a controlled reference system. Therefore, application of polystyrene beads to capture the critical causes at controlled biological system could pave the way to understand the effects of real world NPs. In this work, we have preliminarily showed the protein corona could possible cause coalescence effect in the NPs apart from the protein confirmation changes. Since the prominent proteins in blood are responsible for protein-induced coalescence of NPs, we believe such grain growth in NPs entered in blood stream could possibly block the in- and out-flow of body fluids. On the other hand, the NPs can interrupt mitotic activity and cause cytotoxicity to human and animal cells. This knowledgebase could be useful for acquiring/retrieving the perceptive toxicity information and health risk from real world NPs.

## Supplementary information

Dataset 1
