# Supplementary material for: Assessment on interactive prospectives of nanoplastics with plasma proteins and the toxicological impacts of virgin, coronated and environmentally released-nanoplastics
Source: Sci Rep. 2019 Jun 20;9:8860. doi: 10.1038/s41598-019-45139-6 (PMC6586940; doi:10.1038/s41598-019-45139-6)
Supplement: Supplementary file 1 — Dataset 1 [file 41598_2019_45139_MOESM1_ESM.docx]

**Assessment on interactive prospectives of nanoplastics with plasma proteins and the toxicological impacts of virgin, coronated and environmentally released-nanoplastics**

Gopinath P M, Saranya V, Vijayakumar S, Mythili Meera M, Ruprekha S, Kunal R, Pranay A, John Thomas, Amitava Mukherjee, Chandrasekaran N*

Center for Nanobiotechnology, VIT, Vellore – 632014, TN, India

**Methodology**

**Isolation of nanoplastics from facial scrubs**

Microplastics were collected from commercial facial scrubs via sequential filtration steps (Hernandez, et al., 2017). Briefly, 0.2 g samples were diluted with ultrapure water (10 mL) and subjected to consecutive filtrations using 20-25 μm (Grade 2 Whatman®), 2.5 μm (Grade 41 Whatman®), 0.45 μm (Acrodisc® syringe filter) and 0.1 μm (Acrodisc® syringe filter) filters. Prior to filtration all the filters were rinsed thrice with ultrapure water. The experiment was triplicated. The materials obtained in 0.1 μm filter was dried in a desiccator and the obtained powder was subjected to Fourier Transform Infrared Spectroscopy (FTIR), PerkinElmer 1600 instrument, USA. Virgin-NPs were used as reference sample to confirm the presence of plastic particles. Further, the filtrates were examined under HR-SEM (Carl Zeiss Evo 18 SEM, Germany) by fixing a thin film on copper grid.

**Comet assay**

On a conventional glass slide a layer of normal melting agarose (0.1%) was prepared and dried. About 75 μl of low melting point agarose containing NPs treated lymphocytes was added on the layer, gently covered with clean cove slip and allowed for harden in an ice pack. After hardening the coverslip was removed, then 80 μl of low melting point agarose was added as third layer, coverslip was replaced and placed in the ice pack. Prepared slides were submerged in fresh, cold lysis solution (10 pH; 2.5 M NaCl; 100 mM disodium EDTA; 10 mM Tris base; 1% Triton X-100, 10% DMSO) for at least 2 h at 4° C. Then the slides were gently placed in freshly made ~ pH 13 alkaline buffer (10 N NaOH and 200 mM EDTA-pH 10) for 20 min to unwind the DNA followed by electrophoreses for 30 min at 24 volts. After electrophoresis, the slides were neutralized with 0.4 M Tris (pH 7.5), rinsed twice with cold water and finally stained with EtBr (2μg mL^-1^). The lymphocytotoxic experiment was repeated for all the individual’s lymphocytes with their respective plasma-NPs complex. Additionally, the genotoxicity of virgin-NPs and isolated-NPs were also experimented. The slides were immediately scored using Comet imager version 2.2 (Metasystem, GmbH) mounted in the ZEISS Axio Scope.A1 Fluorescence Microscope (Carl Zeiss, Germany). Thirty cells per replica of three replicates for each NPs concentration from each individual were imaged and analyzed.

***Allium cepa* Assay**

*A. cepa* bulbs were grown in ultrapure water for 2–3 days at room temperature until achieving 2-4 cm root growth. The bulbs were then treated with 5, 10, 15, 20 and 25 μg/mL concentrations of the virgin-NPs and isolated-NPs, separately. Ethylmethane sulfonate (100 µg/mL) and water was used as positive control and negative control, respectively. The root tips from each treatment were collected, fixed in Carnoy’s fixative (acetic acid: alcohol; 1:3 ratio) for 24 h. Then the root tips were washed with ultrapure water, hydrolyzed (1 N HCl at 60–70 °C for 5 min), washed, a thin sections (1-2 mm) of the root tips were made, placed on the microscopic slides and stained (drops) for 2 min with aceto-orcein. Root tips were compressed further with metal rod, stained with aceto-orcein and covered with cover glass. Using Nikon Eclipse TS100 Microscope (USA) the slides were observed chromosome aberrations and photomicro-graphed. About 100 cells per slide were examined for determining mitotic index and chromosomal aberration.

Total Mitotic Index, MI%= $\frac{TDC}{TC} \times100$

Chromosomal Aberration Index, AI%= $\frac{Tabn}{TDC}x100$

Where TDC: total number of dividing cells, TC: total number of cells considered, Tabn : total number of abnormal cells observed.

**Results**

**Isolated-NPs form face scrubs**

SEM micrograph of face scrub filtered using 2.5 μ filter (Fig. S1a) indicates the presence of large irregular particles (~2.5 μm) as well as smaller/nano sized particles (see yellow circle in Fig. S1a) whereas the samples obtained from fourth filtration step (i.e. 0.1 μ filter) displayed uniform particle size (~100 nm) and shape. The samples obtained from 0.1 μ filter was dried and used for FT-IR analysis (Fig. S2) along with the virgin-NPs. Resulting spectra in transmittance from 500 to 4000 cm^-1^ showed strong sp^2^ C-H stretching band between 3000- 3600 cm^-1^ (Jang and Wilkie, 2005), The bands at 2920 and 2850 cm^-1^ are due to the C–H stretching of CH_2_ (Kaewtatip and Tanrattanakul, 2008), the band at ~1630 corroborates the contraction and stretching of C-C bonds in aromatic ring (Wu et al., 2012), framework stretching vibrations of benzene ring was observed at 1460 cm^-1^, and the peak ~910 cm^-1^  is attributed to = C-H bending of the vinyl group in styrene. There are minor spectral bands corresponding to polyethylene particle was also observed in the tested sample. These band patterns are in close correlation with the virgin-NPs marker bands and thus confirmed the presence of NPs in the filtered sample.

**Reference**

Kaewtatip, K., & Tanrattanakul, V. (2008). Preparation of cassava starch grafted with polystyrene by suspension polymerization. *Carbohydrate Polymers*, *73*(4), 647-655.

Hernandez, L. M., Yousefi, N., & Tufenkji, N. (2017). Are there nanoplastics in your personal care products?. *Environmental Science & Technology Letters*, *4*(7), 280-285.

Wu, Y., Song, L., & Hu, Y. (2012). Thermal properties and combustion behaviors of polystyrene/surface-modified TiO2 nanotubes nanocomposites. *Polymer-Plastics Technology and Engineering*, *51*(6), 647-653.

Jang, B. N., & Wilkie, C. A. (2005). The thermal degradation of polystyrene nanocomposite. *Polymer*, *46*(9), 2933-2942.

**Figure S1. HR-SEM micrograph of samples obtained from (a) 2.5 μ and (b) 0.1 μ filters**

**
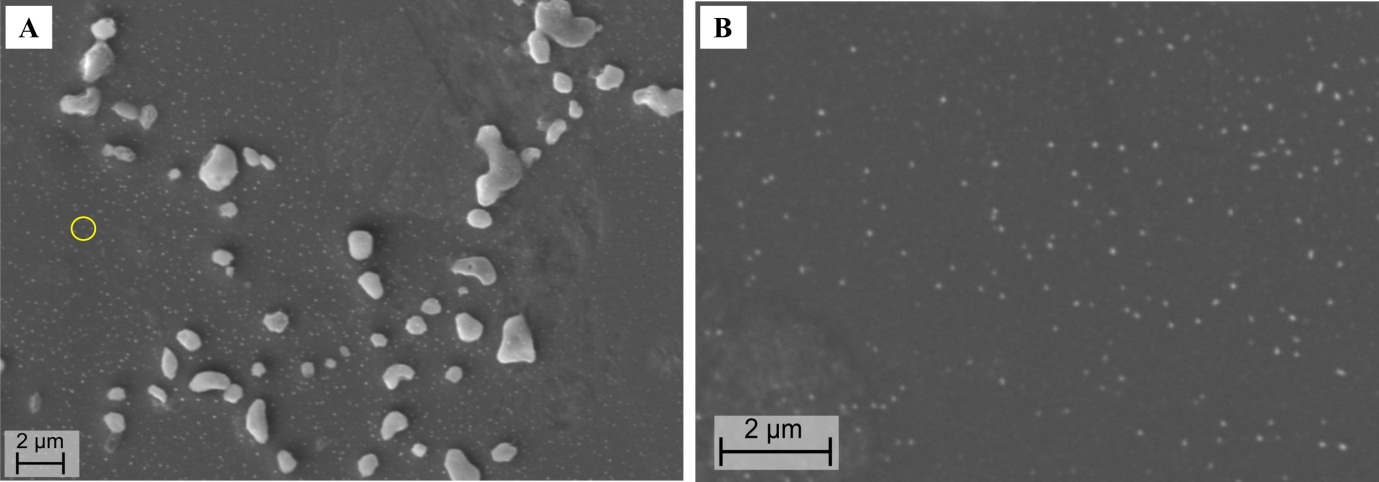
**

**Figure S2. FTIR spectra of NPs obtained from 0.1 μ filter**

**Figure S3. Scanning electron micrograph of coalesced NPs with the grain size of up to 5 μm
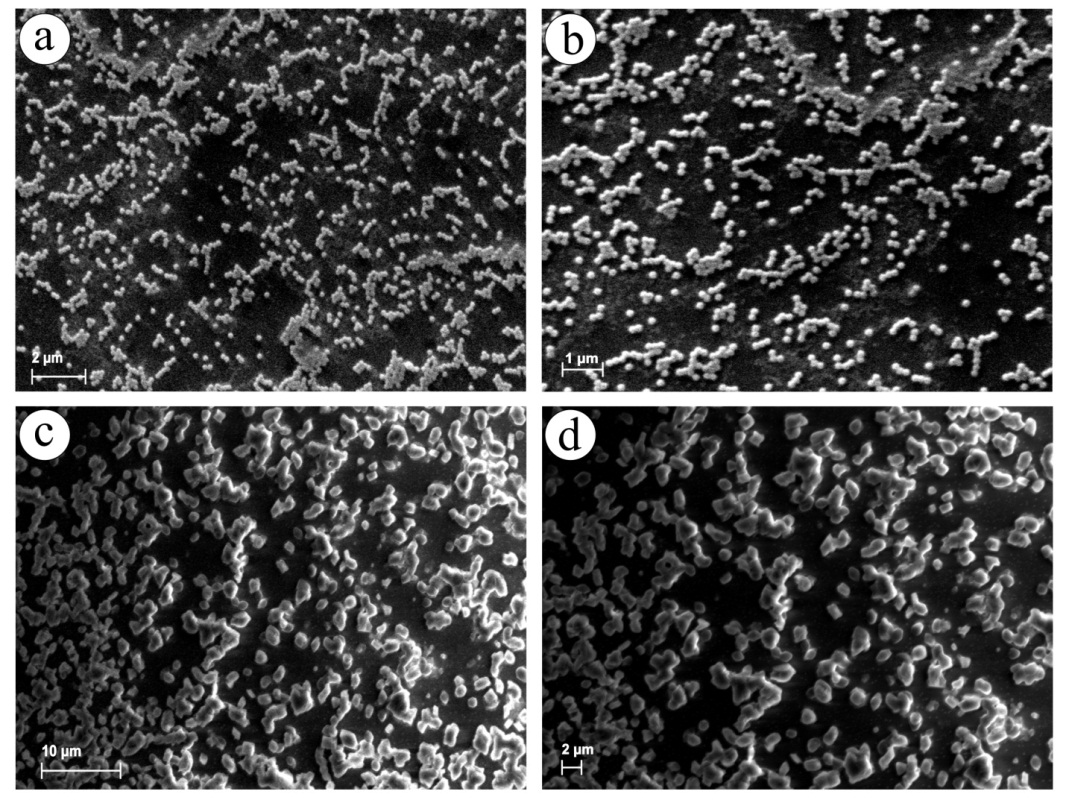
**

**Figure S4. SDS-PAGE analysis of plasma protein conformation upon NPs interaction
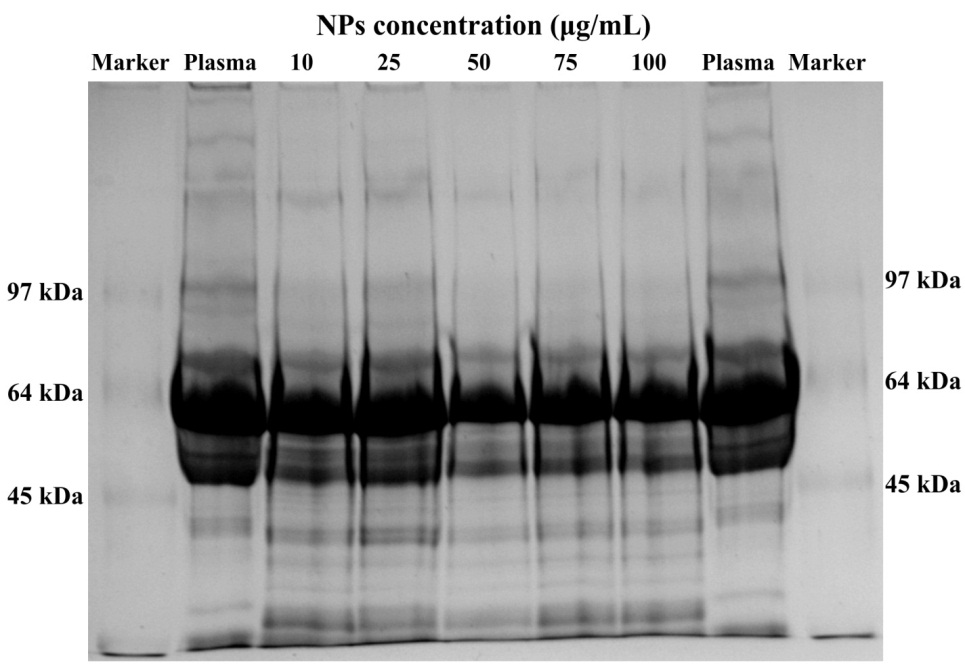
**
